# Engineering and Application of Biosensors for Aromatic Compounds Production in *Escherichia coli*

**DOI:** 10.3390/microorganisms13102358

**Published:** 2025-10-14

**Authors:** Yang Zhao, Pengfei Gu

**Affiliations:** School of Biological Science and Technology, University of Jinan, Jinan 250022, China; 13245313228@163.com

**Keywords:** aromatic compounds, biosensor, *Escherichia coli*, synthetic biology

## Abstract

Aromatic compounds have diverse applications, including flavors, dyes, neurotransmitters, and therapeutics. The microbial fermentation of aromatic compounds from inexpensive substrates is a common strategy; however, optimizing fermentation parameters to enable industrial-scale production remains a major challenge. Biosensors, with their ability to finely tune the expression of endogenous or heterologous pathway enzymes without impeding cell growth, can balance metabolic fluxes and direct them optimally for the synthesis of target products. Thus, biosensor-based strain engineering and screening constitute an intelligent strategy. This article comprehensively summarizes the development and application of aromatic compound biosensors in *E. coli*. Initially, biosensors for aromatic compounds and their working principles of various types of biosensors are reviewed. Subsequently, the latest advancements in these biosensors for engineering microbial cell factories of high-value aromatic compounds are summarized. Finally, the challenges and prospects for constructing robust and sophisticated biosensors for aromatic compounds are discussed. This review can be a valuable reference for constructing diverse biosensors to develop desirable microbial cell factories of aromatic compounds.

## 1. Introduction

Various valuable chemicals are traditionally synthesized based on fossil resources through petrochemical refinery processes. However, excessive use of fossil fuel caused serious climate crisis and environmental issues. In addition, fossil resources will ultimately be depleted. Accordingly, a sustainable production of chemicals from renewable biomass is desirable, such as microbial fermentation. Compared with chemical synthesis, microbial production of chemicals and materials is typically environment-friendly under mild conditions in terms of a relatively low temperature and independent of toxic solvents or metal catalysts. It was reported that the global market size of bio-based chemicals is estimated to reach USD 97.2 billion by 2023, indicating a broad application prospect [1].

Aromatic compounds exhibited diverse application fields, such as flavors, dyes, neurotransmitters, therapeutics and so on. For example, aromatic amino acids including L-tyrosine, L-phenylalanine, and L-tryptophan, are the starting compounds for the biosynthesis of various hormones and neurotransmitters [2]. Recently, a small amount of 4-methoxystyrene (4-vinylanisole), a substance known as migratory locust aggregation pheromone, was successfully synthesized in *E. coli* from glucose by methylating the hydroxyl group of 4-hydroxystyrene [3]. In addition, 5-hydroxytryptophan (5-HTP) has commercial potential in the treatment of various neurological and metabolic diseases, and it is an important precursor of serotonin and melatonin, and it can be converted from L-tryptophan through tryptophan hydroxylase (TPH) [4]. Aromatic compounds can also be employed as building blocks for various chemicals. It was estimated that over 40% of bulk chemicals contained aromatic compounds [5]. Accordingly, the microbial production of aromatic compounds from inexpensive substrates has exhibited increasing opportunities [6]. Traditionally, strains employed in fermentation were predominantly obtained through random mutagenesis followed by selection, a process that was both time-consuming and labor-intensive. Based on genetic and metabolic engineering techniques, various microorganisms with high amino acid production yields are now constructed by rational design of targeted biosynthetic pathways. Several strategies, such as improving the intracellular levels of precursors, controlling the attenuation and feedback inhibition of key enzymes, engineering of global regulators, removing transcriptional suppression, engineering transport system, and decrease of acetate secretion, were employed for constructing aromatic compounds production strains with relatively high titers, yields, and productivities [7,8]. Nevertheless, further enhancement of fermentation parameters to achieve industrial production faces significant challenges.

With the advancement of synthetic and systems biology, a range of innovative technologies has emerged, offering a promising new direction for the engineering of chemical-producing strains, including biosensors [9]. In the context of synthetic biology, biosensors are defined as biological elements capable of converting signals that are not suitable for measurement (such as physical or chemical signals) into directly measurable output signals like gene expression or fluorescence. Initially, various biosensors were employed in the rapid detection of target products. Comparing with the traditional time-consuming and expensive chromatographic methods, they significantly improve the engineering efficiency of microbial cell factories [10].

Traditional static metabolic engineering strategies are commonly used to direct increased carbon flow into the biosynthetic pathways of target compounds. However, static modification of key genes (such as knockout and overexpression) often results in an imbalance within the intracellular metabolic network, as well as the accumulation of intermediates and by-products [11]. Furthermore, real-time monitoring of metabolic fluxes and compounds remains imperfect, thereby limiting the efficiency and precision of metabolic optimization [12]. Dynamic metabolic engineering, in contrast, can regulate gene expression in response to changes in intracellular and extracellular states and environmental conditions, thereby addressing the robustness deficiencies inherent in static control [13,14,15]. In nature, microorganisms precisely regulate their gene expression by monitoring changes in external environmental signals, thereby completing their life cycle from the lag phase to the decline phase. Inspired by this natural dynamic regulatory mechanism, Farmer and Liao, as early as 2000, constructed a dynamic controller that senses the metabolic state of cells and regulates the expression of the lycopene pathway using acetyl-phosphate-responsive transcription factors and their binding promoters (transcription factor-promoter), successfully increasing the yield of lycopene by 50% [14]. This study confirmed that the dynamic control of metabolic flow using metabolite sensors can enhance the yield and production capacity of heterologous pathways.

As biosensors have the capability to finely adjust the expression of endogenous or heterologous pathway enzymes without disrupting cell growth, thereby balancing metabolic fluxes and directing them optimally for the synthesis of target products, biosensor-based strain engineering and screening represent an intelligent strategy [16]. By using specific chemical or signal detection to autonomously regulate the transcription, translation or protein levels of microorganisms, significant changes in metabolic flow can be made, further significantly improving the production of target compounds. This review focuses on the development and application of biosensors in optimizing the production of aromatic compounds in *E. coli* (Table 1). Initially, the biosynthetic pathways of aromatic compounds are elucidated. Subsequently, various biosensors and their applications in the production of aromatic compounds are introduced and analyzed. Furthermore, the developmental prospects and challenges associated with biosensors are also discussed.

## 2. Biosynthetic Pathway of Aromatic Compounds in *E. coli*

**Figure 1:**
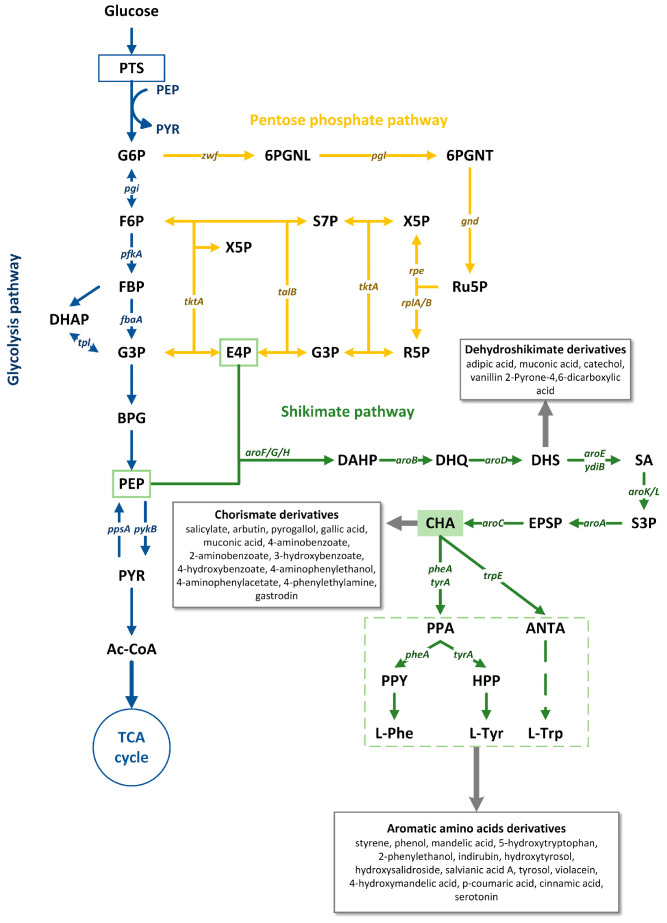
Schematic diagram of biosynthetic pathway of aromatic compounds: PPP pentose phosphate pathway; TCA cycle tricarboxylic acid cycle; G6P glucose-6-phosphate; F6P fructose-6-phosphate; FBP fructose-1,6-biphosphate; G3P glyceraldehde-3-phosphate; BPG 1,3-bisphosphoglycerate; PEP phosphoenolpyruvate; PYR pyruvate; Ru5P ribulose-5-phosphate; R5P ribose-5-phosphate; X5P xylulose-5-phosphate; S7P sedoheptulose-7-phosphate; DHQ 3-dehydroquinate; DHS 3-dehydroshikimate; SA shikimic acid; S3P shikimate-3-phosphate; EPSP 5-enolpyruvylshikimate 3-phosphate; CHA chorismate;PTS phosphoenolpyruvate: carbohydrate phosphotransferase system; TktA transketolase I; Tal transaldolase; PpsA phosphoenolpyruvate synthase; PykF/PykA pyruvate kinase; AroF/AroG/AroH 3-deoxy-D-arabino-heptulosonate-7-phosphate synthase; AroB 3-dehydroquinate synthase; AroD 3-dehydroquinate dehydratase; AroE/YdiB shikimate dehydrogenase; AroK/AroL shikimate kinase; AroA EPSP synthase; and AroC chorismate synthase. Arrows indicate the enzyme reaction directions in the pathway.

The basic metabolic pathways involved in the biosynthesis of aromatic compounds include the glycolytic pathway (Embden–Meyerhof–Parnas pathway, EMP) and the pentose phosphate pathway (PPP), which provide the precursors phosphoenolpyruvate (PEP) and erythrose-4-phosphate (E4P), respectively (Figure 1). And then, PEP and E4P feed into the shikimate pathway, which ultimately leads to the production of aromatic amino acids and other aromatic metabolites [30]. The first step involves the condensation of PEP and E4P to 3-deoxy-D-arabino-heptulosonate-7-phosphate (DAHP) by DAHP synthetase [31]. The DAHP synthase, encoded by genes *aroG*, *aroF*, and *aroH*, represents the first rate-limiting enzyme for shikimic acid pathway. The Dehydroshikimic acid (DHS), as an important intermediate in the shikimic acid pathway, is produced from DAHP through two reactions catalyzed by AroB and AroD, and then, the shikimic acid (SA) is generated from DHS via shikimic acid 5-dehydrogenase (SDH, encoded by *aroE* and *ydiB*) [31]. Subsequently, shikimic acid kinase (SK, encoded by *aroK* and *aroL*) converts SA into S3P (shikimic acid 3-phosphate). The next step is to generate EPSP (5-enolpyruvate shikimic acid 3-phosphate, encoded by *aroA*) [32]. The production of chorismate catalyzed by CS (chorismate synthase, encoded by *aroC*) is the final step of the shikimic acid pathway. In other hand, chorismate is a common precursor for three aromatic amino acids. The initial step in the production of L-phenylalanine and L-tyrosine is catalyzed by chorismate mutase (CM), which converts chorismate into prephenate (PPA). Subsequently, PPA undergoes decarboxylation and dehydration to form phenylpyruvic acid (PPY) or oxidative decarboxylation to yield 4-hydroxyphenylpyruvic acid (HPP). The final step involves the conversion of PPY to L-phenylalanine and 4-HPP to L-tyrosine respectively, catalyzed by aminotransferase (AT). The production of L-tryptophan commences with the catalysis by anthranilate synthase (AS, encoded by the *trpE* gene), which converts chorismate into anthranilate (ANTA), followed by five subsequent reactions to generate L-tryptophan [33,34].

## 3. Construction and Application of Different Biosensors for Aromatic Compounds Production

The fundamental components of different biosensors exhibit significant variation, leading to diverse applications such as the detection of aromatic compound concentrations and high-throughput screening. For example, tryptophan biosensors have significant application value in the dynamic regulation of tryptophan metabolism in cells. The dynamic regulatory system can precisely regulate the metabolic flow and energy flow of cells by inserting regulatory elements into the gene circuit. Compared with the traditional static regulatory strategies (such as gene knockout or overexpression), the dynamic regulation can achieve a better balance between cell growth and product synthesis. The static regulatory strategies, such as directly knocking out the tryptophase gene *tnaA* [35] or overexpressing the key gene *aroG* [36] in the tryptophan synthesis pathway, although can increase the production of tryptophan, they often lead to problems such as cellular metabolic imbalance and accumulation of metabolic by-products. When these by-products reach a certain concentration, bacterial growth may be inhibited, resulting in decreased yields of tryptophan. Therefore, achieving equilibrium between cell growth and aromatic compound production through dynamic regulation of cellular metabolism represents an effective strategy for improving both conversion rates and productivity in aromatic compound synthesis, which can be achieved by different biosensors (Table 2).

### 3.1. TnaC-Based L-Tryptophan Biosensor

The early determination methods of tryptophan mainly relied on chromatography and spectroscopy techniques, such as high performance liquid chromatography (HPLC) [37], ultraviolet spectrophotometry [38], and colorimetry [39]. These methods can achieve precise measurement of tryptophan concentration, but they have limitations such as complex steps and long sample pretreatment time. In recent years, the newly developed tryptophan biosensors have been widely used in the real-time quantitative detection of L-tryptophan concentration [40,41]. The detection range of these biosensors has been continuously expanding and has been successfully applied in the actual production process of tryptophan, gradually replacing the traditional chromatographic and spectroscopic detection methods.

At present, an increasing number of leader peptides have been reported, characterized by specific amino acid sequences. When exogenous small molecules penetrate the cells, they interact with these amino acid sequences, leading to ribosome translation arrest [42]. To modulate gene expression, these leader peptides can be developed into highly specific small molecule sensors [43], such as the tryptophan biosensor pSentrp [17] and TrpSEN [18]. The tryptophanase operon (*tnaCAB*) constitutes a gene cluster responsible for the catabolism of exogenous tryptophan. Within this operon, *tnaA* and *tnaB* serve as structural genes [44,45].

**Figure 2:**
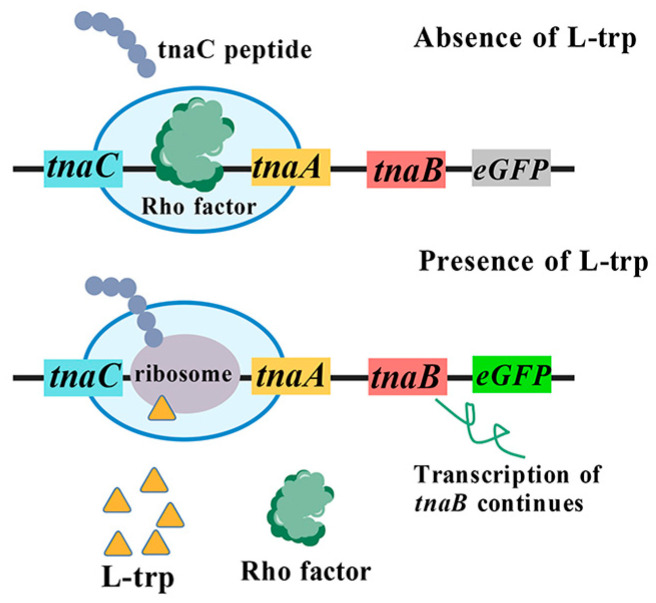
The TnaC-based L-tryptophan biosensor.

The mechanism of the *tnaCAB* operon involves the regulation of ribosome stalling by tryptophan during the translation of the leader peptide TnaC, resulting in transcriptional attenuation of the promoter P_tna_ [46]. When the intracellular concentration of tryptophan is low, the translation of TnaC is effectively terminated by release factor RF2 at its stop codon [47]. Upon completion of TnaC translation, ribosomes dissociate from the mRNA. Subsequently, the Rho factor binds to target site located downstream of the *tnaC* stop codon, thereby obstructing RNA polymerase from associating with P_tna_ and terminating the transcription of downstream genes. Conversely, when tryptophan concentration is high, the release of RF2 is inhibited, resulting in ribosomes remaining bound to the mRNA at *tnaC*. This mechanism inhibits the binding of Rho factor while permitting RNA polymerase to attach to P_tna_, thereby facilitating the normal transcription of downstream genes [45,46,48,49] (Figure 2). Accordingly, *tnaC* can operate as an input sensor that is contingent upon intracellular L-tryptophan concentration, and translate this concentration into signals, such as fluorescence. Fang et al. [17] developed a tryptophan biosensor, pSentrp, utilizing the leader peptide TnaC. In this configuration, pSentrp functions as an intermediate sensor, while L-tryptophan acts both as the product of the upstream module and as the substrate for the downstream module. By integrating both upstream and downstream modules and employing pSentrp as a pivotal component for monitoring tryptophan signals within this metabolic pathway, a flux balance of tryptophan across these pathways was achieved through a “push-pull” mechanism, thereby successfully attaining bidirectional dynamic regulation of tryptophan. Using this system, an *E. coli* cell factory was constructed for the efficient synthesis of the tryptophan derivative deoxyviolacein using glucose, ultimately enhancing the titer of deoxyviolacein by 4.4-fold [17]. The “push-pull” strategy developed by this research can achieve sequential pathway optimization by combining biosensors and key pathway intermediates concentrations, which can be applied in synthesis of several chemicals.

### 3.2. Aptamer-Based L-Tryptophan Biosensor

**Figure 3:**
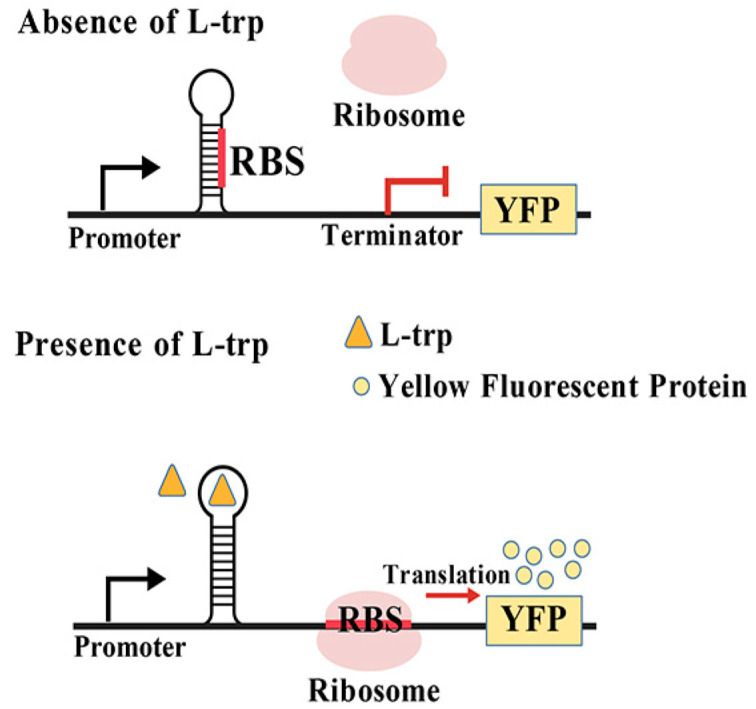
The aptamer-based L-tryptophan biosensor.

The riboswitch is an RNA regulatory element characterized by its ligand-binding “aptamer” domain, which represents its most conserved component. A notable feature of riboswitches is their predominant localization within the 5′ untranslated region (5′ UTR) of the mRNA. This structural arrangement allows the riboswitch to be synthesized first, providing sufficient time for it to respond to metabolite binding before the production of full-length mRNA (or the entire operon), thereby facilitating gene expression regulation at the translational level [50,51]. The riboswitch primarily consists of two domains: the aptamer domain and the expression domain. The aptamer domain is responsible for recognizing and binding small molecule compounds present within the cell, subsequently modulating the activity of the expression domain. In turn, the expression domain regulates downstream gene expression by controlling ribosome binding at specific sites known as ribosome binding sites (RBS) [50] (Figure 3).

In 2012, Yang et al. [19] first constructed a riboswitch-based tryptophan biosensor by selecting (N)10 RNA aptamers, specifically 70–727 (5′-GAGGGTAAGA-3′), which are capable of functionally linking the aptamer region with the selection module (*tetA*-*sgfp*) in vivo [43]. When L-tryptophan is deficient within cells, a stem-loop structure forms at the 5′ UTR of mRNA, thereby obstructing ribosome binding to the RBS, leading to translation termination. Conversely, when there is an excess of L-tryptophan present in cells, L-tryptophan molecules can bind to these stem loops and modify the secondary structure of mRNA. Consequently, the RBS becomes exposed, allowing translation to proceed. Liu et al. [20] developed two activated tryptophan biosensors using two synthetic L-tryptophan aptamers, ribo585 and ribo727, therefore obtaining a high-throughput screening platform based on the riboswitch and fluorescence-activated droplet sorting (FADS) technology. In addition, yellow fluorescence protein (YFP) was selected as reporter gene for the characterization of biosensors in the microfluidic droplets as the index and successfully screened out a mutant strain with significantly increased L-tryptophan titer from the random mutagenesis library. Compared with the control strain KW003, the L-tryptophan titer of this mutant strain increased by 155.1%. In the future, these synthetic RNA devices can be used as a general high-throughput screening platform for accelerating evolution process and obtaining strains with overproduced metabolites.

In addition, Tang et al. [21] combined the tryptophan biosensor R151-GFP developed based on riboswitches with fluorescence activated cell sorting (FACS) and successfully established an efficient and high-throughput screening platform. By using this platform to screen mutant strains derived from multiple generations of atmospheric and room temperature plasma (ARTP), strain Trp01 with L-tryptophan accumulation of 2.15 g/L was obtained in shake flask culture experiment was obtained. By coordinating multi-dimensional engineering with FACS, a microbial cell factory for efficient synthesis of L-tryptophan was established in this study, laying the foundation for industrial production of L-tryptophan and its derivatives.

### 3.3. TF-Based Tryptophan Biosensors

**Figure 4:**
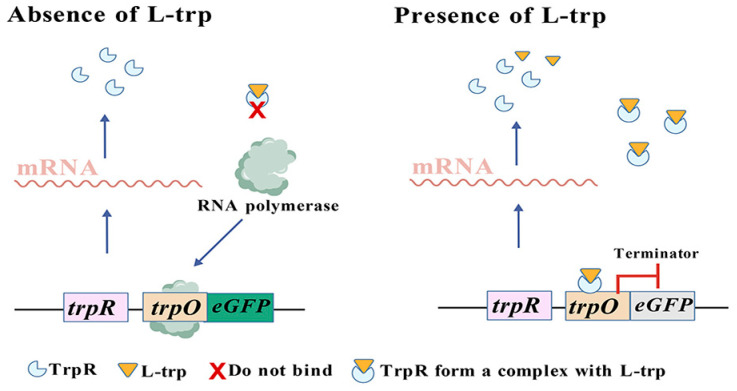
The TF-based tryptophan biosensors.

Among the tryptophan biosensors developed using transcription factors, the tryptophan operon is the most representative system [11]. Its transcription is governed by two mechanisms: repression and attenuation. Presently, the majority of transcription factor-based tryptophan biosensors are primarily developed through the repression mechanism and typically comprise four components: the repressor protein TrpR, the ligand molecule tryptophan, the operator sequence *trpO*, and a reporter gene [22]. Gong et al. [22] constructed the tryptophan biosensor TrpR1PtrpO1 suitable for *E. coli* in 2022. Within the gene regulatory network of *E. coli*, TrpR plays an important regulatory role for numerous genes, including not only the *trpEDCBA* operon [52], but also TrpR itself [53], as well as Mtr [54], which functions as a co-directional transporter for tryptophan transport alongside protons (H^+^). TrpR acts as a dimer repressor, and it can combine with *trpO* located upstream the corresponding manipulation gene and subsequently inhibit the expression of downstream genes when tryptophan is present. Conversely, in the absence of tryptophan, the repressor protein TrpR cannot associate with the regulatory sequence *trpO*, which permits RNA polymerase to bind to the promoter sequence and facilitates normal transcription of downstream genes (Figure 4) [55,56,57]. In addition, the application scope of tryptophan biosensors is not limited to the detection of tryptophan concentration but can also be used to determine the concentration of tryptophan derivatives [58]. The team of Höcker and Jürgens obtained the tryptophan repressor protein TrpR variant TrpR (M42F/T44L/T81M/N87G/S88Y) through amino acid mutation [58]. This variant can stably bind to the tryptophan derivative indole-3-acetic acid (IAA), and conformational changes occur after binding. The fluorescent protein conjugated with it can convey this change, generating and outproducing fluorescence resonance energy transfer (FRET) signals. Based on this principle, the team developed the tryptophan biosensor AuxSen for monitoring IAA concentration, achieving real-time monitoring of auxin concentration within plant cells for the first time [58].

Zhang et al. [23] developed the tryptophan biosensor *trpR_AD_* suitable for yeast in 2020. Their research was based on the *trpR* inhibitor of the *E. coli* tryptophan operon, and they developed the yeast tryptophan biosensor *trpR_AD_*. It is composed of *E. coli* TrpR fusing into the GAL4 activation domain, regulating the *GAL1* promoter of the engineering reporter gene (yeGFP), which includes six copies of TrpR binding sites (*trpO*) upstream of the TATA box (*pGAL1-6x-trpO*) of the *GAL1* promoter [23]. Zhang et al. used high-throughput biosensor screening to train various machine learning algorithms, and the best machine learning-guided design suggestions obtained could increase tryptophan titer and productivity by up to 74% and 43%, respectively. Here, the author efficiently constructs a metabolic pathway design library and uses high-throughput biosensor screening to train various machine learning algorithms, thereby effectively guiding metabolic engineering work.

### 3.4. HucR-V7/PhucR-Based Vanillin Biosensor

**Figure 5:**
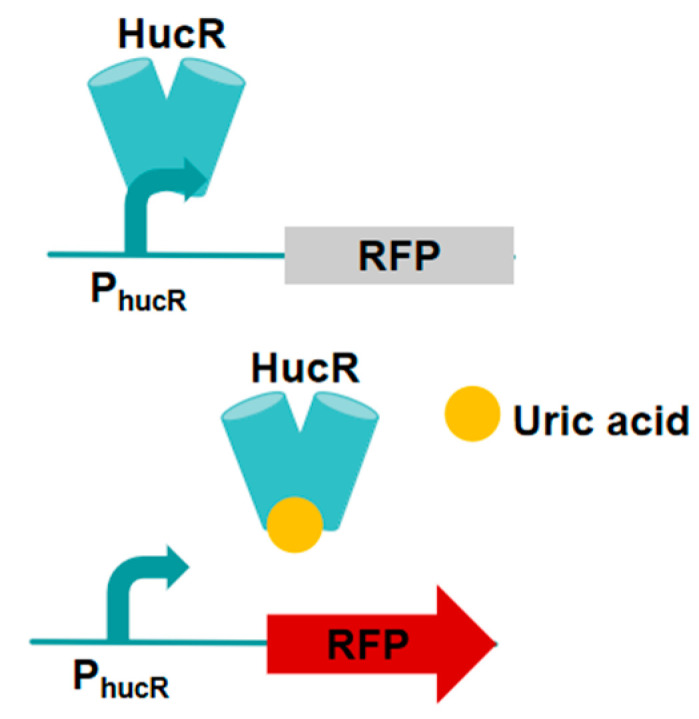
The HucR-V7/PhucR-based vanillin biosensor. The arrows represent P_hucR_ promoter.

Liang et al. [24] engineered a HucR regulatory protein that exhibits responsiveness to uric acid. The hypothetical uricase modulator (HucR) derived from *Deinococcus radiodurans* is classified within the MarR family of transcriptional modulators (Figure 5). This system refers to the fact that in the absence of uric acid, the HucR regulatory proteins can inhibit the gene expression of the promoter PhucR, and the combination of uric acid and HucR induces conformational changes in this protein, leading to the release of HucR promoter DNA and the activation of promoter PhucR transcription [24,59,60]. using this engineered HucR regulatory protein, the researchers investigated the dynamic regulation of the vanillin biosynthesis pathway. In comparison to traditional promoters and quorum sensing promoters, this approach offers significant insights into optimizing the biosynthesis of compounds that may impose additional burdens on host cells or generate toxic products [24]. To modify the effector specificity of HucR and develop regulatory components aligned with the research objectives, Liang et al. [24] identified four amino acid positions within the effector binding pockets that are directly associated with uric acid binding. A site-saturated mutation library of HucR was constructed based on these positions. Subsequently, the library underwent screening using FACS. Liang et al. [24] constructed a site-saturated mutagenesis library of the PhucR promoter −10 region to further increase the yield of vanillin by using cascade dynamic control. Through RFP fluorescence screening, mutant promoters with induction intensities 0.01 to 1.98 times that of wild-type promoters were screened out, thereby enabling better multiple-layer dynamic control of gene transcription in the biosynthetic pathway [24]. This study developed a novel regulatory component through directed evolution to control the vanillin biosynthesis pathway.

### 3.5. PadR/PpadC-Based p-Coumaric Acid Biosensor

**Figure 6:**
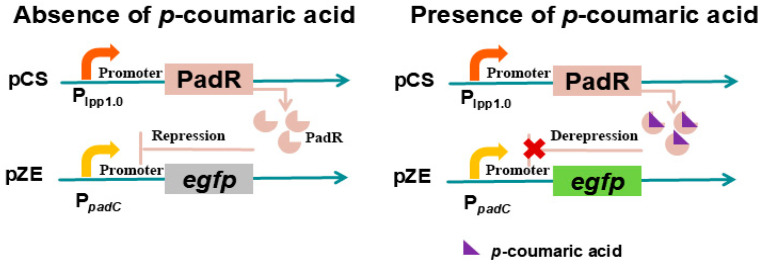
The PadR/PpadC-based *p*-Coumaric acid biosensor.

PadR is a transcriptional repressor derived from *B. subtilis*, which can bind to the −10 region of the *padC* gene promoter PpadC, blocking the entry of RNA polymerase and thereby inhibiting the expression of phenolic acid decarboxylase (padC), thus exerting an inhibitory effect [25]. Upon exposure of *B. subtilis* to environments containing *p*-coumaric acid or ferulic acid, these compounds interact with PadR, inducing conformational changes in the repressor and alleviating its inhibitory effect [61,62]. To facilitate high-throughput screening applications of this system, Haakan N. Joensson et al. [63] precisely calibrated the expression level of PadR through RBS engineering, thereby significantly expanding the dynamic detection range of the PadR-PpadC biosensor (Figure 6). Subsequently, the optimized system was successfully deployed in yeast mutation library, enabling the efficient screening of high-yield *p*-coumaric acid strains, thus verifying the PadR-P_padC_ biosensor system’s capability to screen high-throughput strains with elevated yields of *p*-coumaric acid or ferulic acid [63]. Meanwhile, this system can dynamically regulate the synthesis process of flavonoids and polyphenols derived from these two precursors. Jiang et al. first constructed a functional PadR-P_padC_ biosensor system in *E. coli*, and the constitutive promoter lpp1.0 controls the *padR* gene, while the promoter PpadC cloned from *Bacillus subtilis* controls the reporter gene *egfp* [25]. The results indicated that *p*-coumaric acid successfully activated the biosensor system, but compared with commonly used biosensors, the activation intensity was too low to be applied to dynamic regulation or high-throughput screening [25]. Assuming that the genes *yveF* and *yveG* are in the intergenic region between the P_padC_ and *padC* genes and have a potential impact on the activity of PpadC and the dynamic behavior of the system, these genes were inserted between the PpadC promoter and the egfp gene. A functional analysis and verification indicated that *yveF* and *yveG* significantly enhanced the intensity of PpadC, helping to suppress the release in the PadR-PpadC biosensor system [25].

Raspberry ketone (RK) is a naturally occurring compound derived from raspberry fruits, frequently used as a flavoring agent in the food industry and as an efficacious component for weight loss [64]. Microbial synthesis offers a more efficient and cost-effective approach to the production of RK. Nevertheless, the biosynthesis of RK is impeded by an imbalance in synthetic pathways and a deficiency of precursors, such as tyrosine [65] and malonyl-CoA [66]. In response to this challenge, Zhou et al. [67] employed a PadR/PpadC-based coumaric acid biosensor to dynamically regulate the synthesis and consumption pathways of malonyl-CoA, thereby balancing the metabolic flux distribution between cellular growth and raspberry ketone (RK) biosynthesis [68]. Through the optimization of both the pathway and the fermentation process, the RK yield of engineered strain was significantly enhanced to 415.56 mg/L, representing a 32.4-fold increase. The PadR variants and hybrid promoters obtained in this paper will expand the applicability of this sensor system in future metabolic engineering research.

### 3.6. TtgR-Based (2S)-Naringenin Biosensor

**Figure 7:**
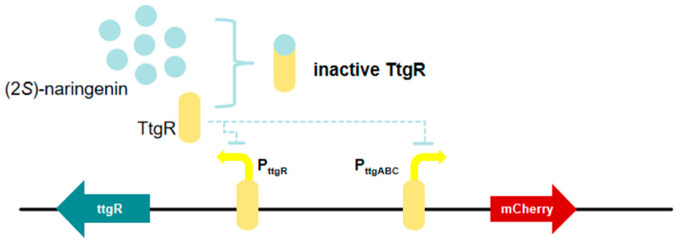
The TtgR-based (2*S*)-naringenin biosensor. The yellow arrows of P_ttgR_ and P_ttgABC_ represent promoter.

Chalcone synthase (CHS) catalyzes the rate-limiting step in the biosynthesis of (2*S*)-naringenin, which serves as the fundamental structure of flavonoids [69]. Enhancing CHS activity through protein engineering is anticipated to substantially increase the yield of (2*S*)-naringenin synthesized by microbial cell factories, thereby facilitating the production of valuable flavonoids [70]. Tong et al. [26] developed a (2S)-naringenin biosensor based on the TtgR operon in *E. coli* (Figure 7). Through promoter optimization, they expanded the detection range to 0–300 mg/L, representing the broadest detection range of (2*S*)-naringenin biosensor reported to date. In addition, this biosensor was specific to (2*S*)-naringenin and was not significantly influenced by precursors or naringenin analogs, rendering it suitable for high-throughput screening of mutants. As a result, (2*S*)-naringenin biosensors based high-throughput screening is an effective method for identifying CHS mutants with high catalytic activity. FapR is a transcriptional suppressor derived from *B. subtilis*, and its ligand is malonyl-CoA [71]. Existing studies have reported that TtgR is a reporter inhibitor derived from *Pseudomonas putida* and is a member of the transcription inhibitor TetR family [72]. It can bind to different antibiotics, flavonoids and organic solvents to regulate the TtgABC effloressor pump [73]. Apart from TtgR, FdeR and riboswitch can also be applied for constructing (2*S*)-naringenin biosensors, and the maximum detection limits of FdeR and riboswitch for (2*S*)-naringenin, as reported by Xiong et al. [74] were 50 and 100 mg/L respectively, which were both lower than that of TtgR. These findings will facilitate efficient flavonoid biosynthesis and further modification of the CHS in the future.

### 3.7. MuYqhC-Based Vanillin Biosensor

**Figure 8:**
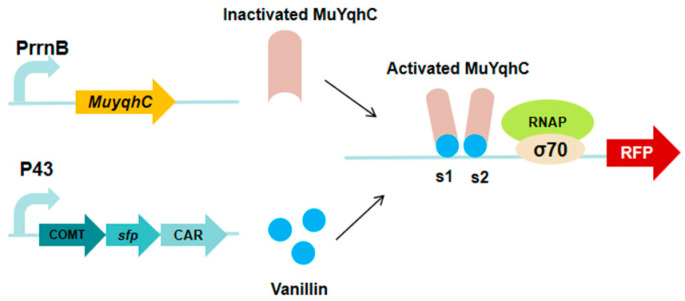
The MuYqhC-based vanillin biosensor. The arrows in the figure indicate the reaction direction.

O-methyltransferase (caffeate O-methyltransferase, COMT) has been identified as the rate-limiting enzyme in the vanillin biosynthetic pathway [17]. The low activity of COMT, coupled with the requirement for the cofactor S-adenosylmethionine (SAM), significantly impedes the production yield of vanillin [75]. YqhC, an aldehyde-responsive transcriptional modulator from the AraC family, is involved in the regulation of the *yghD-dgkA* operon [76,77,78,79]. Dong et al. [27] developed a dual-response biosensor from this component and established a vanillin biosensing system compatible with the COMT-mediated biosynthetic pathway (Figure 8). This study devised a pathway adaptation strategy based on vanillin biosensors and evolved to obtain COMT variants with superior performance accordingly. Using this biosensor, the authors rapidly identified the COMT variant Mu176 from the mutation library, which exhibits a conversion efficiency seven times greater than that of the wild type in catalytically generating vanillin. Subsequently, Mu176 was introduced into the shikimic acid chassis of *B. subtilis*. By optimizing the intracellular supply of cofactors and precursors, the vanillin yields ultimately reached 1 mM, surpassing the highest level publicly reported in *Bacillus* sp. This work highlights the significance of the biosensing system based on MuYqhC and the Mu176 variant in vanillin production.

### 3.8. Protein Translation Elements Based Amino Acids Biosensor

This category includes biosensors that are developed based on elements of protein translation. For example, Guo et al. [28] concentrated on creating tryptophan biosensors by employing rare codons. By altering the tRNA anticodon to CUA—where CUA corresponds to the anticodon of the tryptophan non-coding codon UAG—the researchers noted a decrease in affinity for aminoacyl-tRNA synthetases (aaRS). A screening system was constructed for marker genes containing the UAG codon along with the corresponding tRNA^Trp^_CUA_. High-yield L-tryptophan strains were successfully isolated from a mutant library of *E. coli*. Notably, strain TP2 achieved an L-tryptophan yield of 8.9 mg/L, which is 2.3-fold greater than that of the wild type, thereby validating this screening strategy [28].

**Figure 9:**
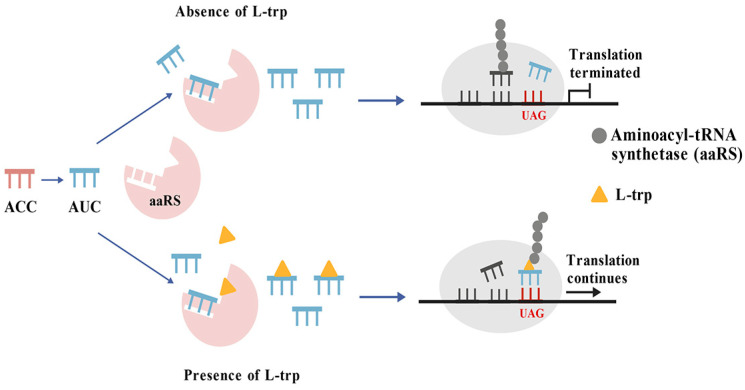
The Protein translation elements based amino acids biosensor. The arrows in the figure indicate the reaction direction.

Concurrently, a mutation was introduced into the kanamycin resistance gene, *kanR*, wherein its tryptophan codon was altered to UAG. At this point, the translation of the UAG codon became dependent on the availability of aminoacyl-tRNA^Trp^_CUA_ [28]. In the absence of exogenous tryptophan, the synthesis of aminoacyl-tRNA^Trp^_CUA_ is restricted. As a result, *kanR* cannot be adequately expressed, and cells fail to grow normally on kanamycin-containing plates. Conversely, when exogenous tryptophan is provided, normal synthesis of aminoacyl-tRNA^Trp^_CUA_ occurs, along with an excess supply that facilitates the sufficient formation of aaRS-Trp complexes [80,81]. Under these conditions, *kanR* expression resumes normally, and cellular growth returns to baseline levels. Consequently, this novel tryptophan biosensor, utilizing the rare codon UAG, effectively translates intracellular tryptophan concentrations into a readily observable cell growth rate (Figure 9). Guo et al. [28] innovatively employed tryptophan biosensors derived from protein translation elements to develop an effective screening system for high-yield tryptophan strains. Initially, they used a random mutation library of *E. coli* and, through dual screening methods involving strain growth and FACS, successfully identified a mutant with a significantly enhanced L-tryptophan titer, which was 1.3-fold greater than that of the original strain. Building on this foundation, the production potential of the strain was further investigated through metabolic engineering optimization, ultimately achieving a tryptophan yield of 20.3 g/L. Here, the author developed a novel selection system that uses the corresponding engineered tRNAs and marker genes to select the overproducers of specific amino acids.

### 3.9. Enzyme-Coupled Tryptophan Biosensors

**Figure 10:**
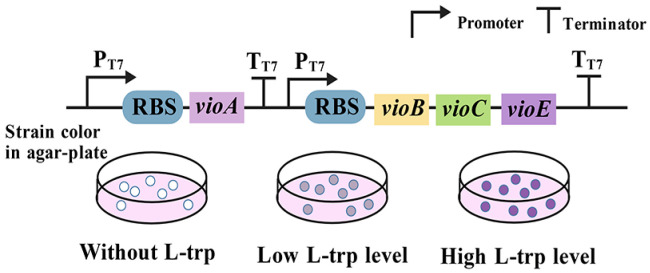
The VioA-based L-tryptophan biosensor.

These biosensors incorporate an enzyme that catalyzes a reaction to convert a specific target metabolite into a readily detectable product, such as a color change or fluorescence [82]. Violacein is synthesized through a five-step reaction involving two molecules of L-tryptophan as substrates, catalyzed by the VioABCDE enzyme complex [83], resulting in the bright purple by-product deoxyviolacein. Subsequent studies [84] demonstrated that the synthesis of deoxyviolacein requires only the participation of VioABCE. Using this property, Li et al. conducted an extensive characterization of the biosynthetic pathway for violacein in *E. coli*, utilizing an L-tryptophan biosensor [29]. Optimization of the first enzyme, VioA, was achieved through RBS engineering and testing various strains expressing VioA to create an L-tryptophan biosensor with a dynamic range extending up to 55-fold (Figure 10). In addition, this sensor can detect extracellularly supplied L-tryptophan concentrations ranging from 0 to 10 g/L [20]. Despite the nascent stage of enzyme-coupled tryptophan biosensor development, these sensors are relatively straightforward to construct due to their dependence on a specific enzyme-catalyzed reaction, which imparts a high degree of specificity. Nevertheless, several challenges persist that require resolution: the high cost associated with these sensors, reduced stability due to the enzyme’s susceptibility to inactivation, operational constraints at a temperature of only 30 °C, and variable sensitivities to tryptophan contingent upon the source of VioA [82].

## 4. Conclusions and Prospects

Biosensors for aromatic compounds have a broad spectrum of significant applications within the field of synthetic biology. In the context of strain screening and evolution, the integration of biosensors for aromatic compounds with FACS proves to be highly effective. Consequently, it enables the efficient and precise identification of strains with superior traits, thereby establishing a robust foundation and providing substantial support for the efficient production of aromatic compounds. Additionally, biosensors for aromatic compounds facilitate real-time quantitative assessment of the growth status of target strains and the yield of aromatic compounds. This precise detection method offers critical data support for strain optimization and the precise regulation of the synthetic pathway. Regarding the regulation of metabolic pathways, biosensors for aromatic compounds can be effectively integrated with other biological components to establish a dynamically regulated system.

Biosensors for aromatic compounds have increasingly attracted scholarly attention and are important in the development of efficient production strains. Critical parameters such as specificity, sensitivity, operating range, and dynamic range are essential in assessing the suitability of a biosensor for practical applications [85]. However, numerous previously reported biosensors for aromatic compounds do not satisfy these stringent criteria. The computer-aided biological design automation of biosensors represents a promising avenue for future development. For instance, Lim et al. used machine learning methods to clarify the transcriptional regulatory network at the genomic scale of *P. putida* KT2440, providing us with a clearer understanding of the regulatory functions of 39 independent regulatory genes related to transcription factors [86]. Maybe this strategy can also be applied in *E. coli*. With development of technologies such as metabolic modeling, machine learning provides a promising tool for guiding metabolic engineering through a learning model based on a systematic analysis of large experimental datasets [87,88]. The combined development of mechanistic and machine learning models enables accurate genotype-to-phenotype prediction, which can be further applied to the engineering and optimization of aromatic compounds biosynthesis in the future [23]. In the field of metabolic engineering in the future, biosensors can be used to assist in high-throughput screening to sense target chemicals or major precursors and discover or develop new or engineered enzymes. These enzymes can be used to enhance the rate-limiting steps in existing production pathways or create an entirely new one, thereby achieving efficient microbial cell factories with economic viability [89,90,91,92,93,94]. At present, the number of transcription factors available for biosensors is relatively small, the response threshold to metabolites is narrow, the regulatory range is limited, and the regulatory time is uncertain, which still restricts the effective application of these strategies in metabolic engineering [95]. By combining the dynamic regulatory strategies at the translation and post-translation levels with the dynamic regulatory system at the transcriptional level [96], more suitable and effective microbial cells can be constructed. This enables a more refined coordinated regulation of metabolic pathways, which can effectively increase the yield and conversion efficiency of the target product.

Furthermore, the universality of biosensors for aromatic compounds across different hosts remains a significant challenge. While biosensors may function effectively in one host, a new design-build-test cycle is necessary when the host is altered. It is important to develop universal regulatory elements for biosensors for aromatic compounds that maintain stability and functionality across various recombinant cells. With advancements in machine learning, models capable of predicting and reconstructing the desired biosensor in a specific host can now be successfully developed [16,97]. In conclusion, in the future, research can focus on building cross-species machine learning models based on different microbial databases, which can become a powerful tool for guiding the reconstruction of universal regulatory elements.

## Figures and Tables

**Table 1:** Characteristics of different aromatic compounds biosensors.

Name	Output Signal	Basic Elements	Advantages	References
TnaC-based L-tryptophan biosensor	Enhanced Green Fluorescent Protein (eGFP)	pSensor, TrpSEN	High specificity, wide range of applications	[17,18]
Aptamer-based L-tryptophan biosensor	Tetracycline efflux pump encoded gene *tetA*; GFP; Yellow Fluorescent Protein (YFP);	Tryptophan Riboselector, p15-ribo585, p15-ribo727, pUC19-R151-GFP	Fast response of L-Tryptophan, high specificity and sensitivity	[19,20,21]
TF-based tryptophan biosensors	Enhanced Green Fluorescent Protein (eGFP); Yeast-enhanced Green Fluorescent Protein (yeGFP)	TrpR1-PtrpO1, pGAL1-6x-trpO	High dynamic range	[22,23]
HucR-V7/PhucR-based vanillin biosensor	Red Fluorescent Protein (RFP)	pPhucR-RFP	Introduce feedback activation and cascading dynamic control strategies	[24]
PadR/PpadC-based *p*-Coumaric acid biosensor	eGFP	pCS-lpp1.0-*egfp*, pZE-PpadC-*egfp*	Increased dynamic range and superior sensitivity	[25]
TtgR-based (2S)-naringenin biosensor	Monomeric Cherry Red Fluorescent Protein (mCherry)	pPttgR-ttgR, pPttgABC-mCherry	The widest detection range for (2S)-naringenin	[26]
MuYqhC-based vanillin biosensor	RFP	pPrrnB-MuYqhC	Screened out MuYqhC, and established a dual-responsive biosensing system	[27]
Protein translation elements based amino acids biosensor	*Kanamycin resistance gene* (*KanR*)	ptRNA^Trp^_CUA_	High specificity	[28]
Enzyme-coupled tryptophan biosensors	Strain color	VioABCDE enzyme complex	High specificity, easy to be engineered	[29]

**Table 2:** Application of biosensors in microbial production of aromatic compounds.

Biosensors	Sensing Signal	Performance Improvement	Reference
pSensor	L-tryptophan	Increasing the deoxyviolacein titer by 4.4-fold	[17]
TrpSEN	L-tryptophan	Increasing the violacein titer by 2.7-fold	[18]
p15-ribo727	L-tryptophan	Obtaining a mutant strain with 155.1% higher L-tryptophan titer	[20]
pUC19-R151-GFP	L-tryptophan	The yield of the screening strain was increased by 4.7-fold after modification	[21]
pGAL1-6x-trpO	L-tryptophan	Increasing L-tryptophan titer and productivity by up to 74% and 43% respectively	[23]
pZE-PpadC-*egfp*	*p*-Coumaric acid	The raspberry ketone yield enhanced to 415.56 mg/L, representing a 32.4-fold increase	[25]
pPttgABC-mCherry	(2*S*)-naringenin	Increasing the (2*S*)-naringenin titer by 65.34%	[26]
pPrrnB-MuYqhC	Vanillin	The titer increased by 2.39-fold, vanillin yields ultimately reached 1 mM	[27]
ptRNA^Trp^_CUA_	L-tryptophan	Increasing the L-tryptophan titer by 1.3-fold, achieving the L-tryptophan titer to 20.3 g/L	[28]

## Data Availability

No datasets were generated or analyzed during the current study.

## References

[1] Ko Y.S., Kim J.W., Lee J.A., Han T., Kim G.B., Park J.E., Lee S.Y. (2020). Tools and strategies of systems metabolic engineering for the development of microbial cell factories for chemical production. Chem. Soc. Rev..

[2] Hirasawa T., Satoh Y., Koma D. (2025). Production of aromatic amino acids and their derivatives by *Escherichia coli* and *Corynebacterium glutamicum*. World J. Microbiol. Biotechnol..

[3] Hu C., Yang J., Guo W., Pan H., Guo D. (2024). De Novo Biosynthesis of 4-Vinylanisole in Engineered *Escherichia coli*. J. Agric. Food Chem..

[4] Maffei M.E. (2020). 5-Hydroxytryptophan (5-HTP): Natural Occurrence, Analysis, Biosynthesis, Biotechnology, Physiology and Toxicology. Int. J. Mol. Sci..

[5] Dickey R.M., Forti A.M., Kunjapur A.M. (2021). Advances in engineering microbial biosynthesis of aromatic compounds and related compounds. Bioresour. Bioprocess..

[6] Dhande Y.K., Xiong M., Zhang K. (2012). Production of C5 carboxylic acids in engineered *Escherichia coli*. Process Biochem..

[7] Kang N.K., Baek K., Koh H.G., Atkinson C.A., Ort D.R., Jin Y.S. (2022). Microalgal metabolic engineering strategies for the production of fuels and chemicals. Bioresour. Technol..

[8] Volk M.J., Tran V.G., Tan S.I., Mishra S., Fatma Z., Boob A., Li H., Xue P., Martin T.A., Zhao H. (2023). Metabolic Engineering: Methodologies and Applications. Chem. Rev..

[9] Liu D., Evans T., Zhang F. (2015). Applications and advances of metabolite biosensors for metabolic engineering. Metab. Eng..

[10] Teng Y., Zhang J., Jiang T., Zou Y., Gong X., Yan Y. (2022). Biosensor-enabled pathway optimization in metabolic engineering. Curr. Opin. Biotechnol..

[11] Mitchler M.M., Garcia J.M., Montero N.E., Williams G.J. (2021). Transcription factor-based biosensors: A molecular-guided approach for natural product engineering. Curr. Opin. Biotechnol..

[12] Machado D., Costa R.S., Ferreira E.C., Rocha I., Tidor B. (2012). Exploring the gap between dynamic and constraint-based models of metabolism. Metab. Eng..

[13] Boada Y., Vignoni A., Picó J., Carbonell P. (2020). Extended Metabolic Biosensor Design for Dynamic Pathway Regulation of Cell Factories. Iscience.

[14] Farmer W.R., Liao J.C. (2000). Improving lycopene production in *Escherichia coli* by engineering metabolic control. Nat. Biotechnol..

[15] Tan S.Z., Prather K.L. (2017). Dynamic pathway regulation: Recent advances and methods of construction. Curr. Opin. Chem. Biol..

[16] Yu W., Xu X., Jin K., Liu Y., Li J., Du G., Lv X., Liu L. (2023). Genetically encoded biosensors for microbial synthetic biology: From conceptual frameworks to practical applications. Biotechnol. Adv..

[17] Fang M., Wang T., Zhang C., Bai J., Zheng X., Zhao X., Lou C., Xing X.H. (2016). Intermediate-sensor assisted push-pull strategy and its application in heterologous deoxyviolacein production in *Escherichia coli*. Metab. Eng..

[18] Gwon D.A., Seok J.Y., Jung G.Y., Lee J.W. (2021). Biosensor-Assisted Adaptive Laboratory Evolution for Violacein Production. Int. J. Mol. Sci..

[19] Yang J., Seo S.W., Jang S., Shin S.I., Lim C.H., Roh T.Y., Jung G.Y. (2013). Synthetic RNA devices to expedite the evolution of metabolite-producing microbes. Nat. Commun..

[20] Liu Y., Yuan H., Ding D., Dong H., Wang Q., Zhang D. (2021). Establishment of a Biosensor-based High-Throughput Screening Platform for Tryptophan Overproduction. ACS Synth. Biol..

[21] Tang M., Pan X., Yang T., You J., Zhu R., Yang T., Zhang X., Xu M., Rao Z. (2023). Multidimensional engineering of *Escherichia coli* for efficient synthesis of L-tryptophan. Bioresour. Technol..

[22] Gong X., Zhang R., Wang J., Yan Y. (2022). Engineering of a TrpR-Based Biosensor for Altered Dynamic Range and Ligand Preference. ACS Synth. Biol..

[23] Zhang J., Petersen S.D., Radivojevic T., Ramirez A., Pérez-Manríquez A., Abeliuk E., Sánchez B.J., Costello Z., Chen Y., Fero M.J. (2020). Combining mechanistic and machine learning models for predictive engineering and optimization of tryptophan metabolism. Nat. Commun..

[24] Liang C., Zhang X., Wu J., Mu S., Wu Z., Jin J.M., Tang S.Y. (2020). Dynamic control of toxic natural product biosynthesis by an artificial regulatory circuit. Metab. Eng..

[25] Jiang T., Li C., Yan Y. (2021). Optimization of a p-Coumaric Acid Biosensor System for Versatile Dynamic Performance. ACS Synth. Biol..

[26] Tong Y., Li N., Zhou S., Zhang L., Xu S., Zhou J. (2024). Improvement of Chalcone Synthase Activity and High-Efficiency Fermentative Production of (2S)-Naringenin via In Vivo Biosensor-Guided Directed Evolution. ACS Synth. Biol..

[27] Dong P., Fan Y., Huo Y.X., Sun L., Guo S. (2024). Pathway-Adapted Biosensor for High-Throughput Screening of O-Methyltransferase and its Application in Vanillin Synthesis. ACS Synth. Biol..

[28] Guo H., Wang N., Ding T., Zheng B., Guo L., Huang C., Zhang W., Sun L., Ma X., Huo Y.X. (2023). A tRNA Modification-based strategy for Identifying amino acid Overproducers (AMINO). Metab. Eng..

[29] Li R., Zhang L., Liu C., Liu X., Bai Z., Yang Y., Li Y. (2023). Development of an L-tryptophan biosensor based on the violacein biosynthesis pathway. Biotechnol. Bull..

[30] Mir R., Jallu S., Singh T.P. (2015). The shikimate pathway: Review of amino acid sequence, function and three-dimensional structures of the enzymes. Crit. Rev. Microbiol..

[31] Li M., Liu C., Yang J., Nian R., Xian M., Li F., Zhang H. (2020). Common problems associated with the microbial productions of aromatic compounds and corresponding metabolic engineering strategies. Biotechnol. Adv..

[32] Herrmann K.M., Weaver L.M. (1999). The Shikimate Pathway. Annu. Rev. Plant Biol..

[33] Priya V.K., Sarkar S., Sinha S. (2014). Evolution of tryptophan biosynthetic pathway in microbial genomes: A comparative genetic study. Syst. Synth. Biol..

[34] Tzin V., Galili G., Aharoni A. (2012). The shikimate pathway and aromatic amino Acid biosynthesis in plants. Annu. Rev. Plant Biol..

[35] Gu P., Yang F., Kang J., Wang Q., Qi Q. (2012). One-step of tryptophan attenuator inactivation and promoter swapping to improve the production of L-tryptophan in *Escherichia coli*. Microb. Cell Factories.

[36] Ding D., Bai D., Li J., Mao Z., Zhu Y., Liu P., Lin J., Ma H., Zhang D. (2021). Analyzing the genetic characteristics of a tryptophan-overproducing *Escherichia coli*. Bioprocess. Biosyst. Eng..

[37] Patrick J.S., Lagu A.L. (1991). A method for the quantitation of tryptophan in *Escherichia coli* fermentation broth by isocratic high-performance liquid chromatography with ultraviolet detection. Anal. Biochem..

[38] Delhaye S., Landry J. (1986). High-performance liquid chromatography and ultraviolet spectrophotometry for quantitation of tryptophan in barytic hydrolysates. Anal. Biochem..

[39] Gruen L.C., Rivett D.E. (1971). Specificity of Spies and Chambers reagent for colorimetric estimation of tryptophan. Anal. Biochem..

[40] Duan X., Chen Z., Tang S., Ge M., Wei H., Guan Y., Zhao G. (2020). A Strategy Employing a TF-Splinting Duplex Nanoswitch to Achieve Single-Step, Enzyme-Free, Signal-On Detection of l-Tryptophan. ACS Sens..

[41] Jang S., Jung G.Y. (2018). Systematic optimization of L-tryptophan riboswitches for efficient monitoring of the metabolite in *Escherichia coli*. Biotechnol. Bioeng..

[42] Ito K., Chiba S. (2013). Arrest peptides: Cis-acting modulators of translation. Annu. Rev. Biochem..

[43] Majerfeld I., Yarus M. (2005). A diminutive and specific RNA binding site for L-tryptophan. Nucleic Acids Res..

[44] Deeley M.C., Yanofsky C. (1981). Nucleotide sequence of the structural gene for tryptophanase of *Escherichia coli* K-12. J. Bacteriol..

[45] Gong F., Yanofsky C. (2002). Instruction of translating ribosome by nascent peptide. Science.

[46] Stewart V., Yanofsky C. (1985). Evidence for transcription antitermination control of tryptophanase operon expression in *Escherichia coli* K-12. J. Bacteriol..

[47] San Martín A., Ceballo S., Baeza-Lehnert F., Lerchundi R., Valdebenito R., Contreras-Baeza Y., Alegría K., Barros L.F. (2014). Imaging mitochondrial flux in single cells with a FRET sensor for pyruvate. PLoS ONE.

[48] Gong F., Yanofsky C. (2001). Reproducing tna operon regulation in vitro in an S-30 system. Tryptophan induction inhibits cleavage of TnaC peptidyl-tRNA. J. Biol. Chem..

[49] van der Stel A.X., Gordon E.R., Sengupta A., Martínez A.K., Klepacki D., Perry T.N., Herrero Del Valle A., Vázquez-Laslop N., Sachs M.S., Cruz-Vera L.R. (2021). Structural basis for the tryptophan sensitivity of TnaC-mediated ribosome stalling. Nat. Commun..

[50] Breaker R.R. (2011). Prospects for riboswitch discovery and analysis. Mol. Cell.

[51] Findeiß S., Etzel M., Will S., Mörl M., Stadler P.F. (2017). Design of Artificial Riboswitches as Biosensors. Sensors.

[52] Kumamoto A.A., Miller W.G., Gunsalus R.P. (1987). *Escherichia coli* tryptophan repressor binds multiple sites within the *aroH* and *trp* operators. Genes Dev..

[53] Bogosian G., Somerville R.L., Nishi K., Kano Y., Imamoto F. (1984). Transcription of the *trpR* gene of *Escherichia coli*: An autogeneously regulated system studied by direct measurements of mRNA levels in vivo. Mol. Gen. Genet..

[54] Jeeves M., Evans P.D., Parslow R.A., Jaseja M., Hyde E.I. (1999). Studies of the *Escherichia coli* Trp repressor binding to its five operators and to variant operator sequences. Eur. J. Biochem..

[55] Bass S., Sugiono P., Arvidson D.N., Gunsalus R.P., Youderian P. (1987). DNA specificity determinants of *Escherichia coli* tryptophan repressor binding. Genes Dev..

[56] Gunsalus R.P., Yanofsky C. (1980). Nucleotide sequence and expression of *Escherichia coli trpR*, the structural gene for the trp aporepressor. Proc. Natl. Acad. Sci. USA.

[57] Zhao D., Arrowsmith C.H., Jia X., Jardetzky O. (1993). Refined solution structures of the *Escherichia coli* trp holo- and aporepressor. J. Mol. Biol..

[58] Herud-Sikimić O., Stiel A.C., Kolb M., Shanmugaratnam S., Berendzen K.W., Feldhaus C., Höcker B., Jürgens G. (2021). A biosensor for the direct visualization of auxin. Nature.

[59] Wilkinson S.P., Grove A. (2004). HucR, a novel uric acid-responsive member of the MarR family of transcriptional regulators from Deinococcus radiodurans. J. Biol. Chem..

[60] Wilkinson S.P., Grove A. (2005). Negative cooperativity of uric acid binding to the transcriptional regulator HucR from *Deinococcus radiodurans*. J. Mol. Biol..

[61] Nguyen T.K., Tran N.P., Cavin J.F. (2011). Genetic and biochemical analysis of PadR-*padC* promoter interactions during the phenolic acid stress response in *Bacillus subtilis* 168. J. Bacteriol..

[62] Tran N.P., Gury J., Dartois V., Nguyen T.K., Seraut H., Barthelmebs L., Gervais P., Cavin J.F. (2008). Phenolic acid-mediated regulation of the *padC* gene, encoding the phenolic acid decarboxylase of *Bacillus subtilis*. J. Bacteriol..

[63] Siedler S., Khatri N.K., Zsohár A., Kjærbølling I., Vogt M., Hammar P., Nielsen C.F., Marienhagen J., Sommer M.O.A., Joensson H.N. (2017). Development of a Bacterial Biosensor for Rapid Screening of Yeast p-Coumaric Acid Production. ACS Synth. Biol..

[64] Tan K.H., Nishida R. (2024). A review on natural phenylbutanoid attractants: Occurrence, distribution, and role in nature, especially in relation to Dacini fruit fly behavior and pollination. J. Chem. Ecol..

[65] Koopman F., Beekwilder J., Crimi B., van Houwelingen A., Hall R.D., Bosch D., van Maris A.J., Pronk J.T., Daran J.M. (2012). De novo production of the flavonoid naringenin in engineered *Saccharomyces cerevisiae*. Microb. Cell Factories.

[66] Peng H., Chen R., Shaw W.M., Hapeta P., Jiang W., Bell D.J., Ellis T., Ledesma-Amaro R. (2023). Modular Metabolic Engineering and Synthetic Coculture Strategies for the Production of Aromatic Compounds in Yeast. ACS Synth. Biol..

[67] Zhou S., Zhang Q., Yuan M., Yang H., Deng Y. (2024). Static and Dynamic Regulation of Precursor Supply Pathways to Enhance Raspberry Ketone Synthesis from Glucose in *Escherichia coli*. J. Agric. Food Chem..

[68] Zhou P., Fang X., Xu N., Yao Z., Xie W., Ye L. (2021). Development of a Highly Efficient Copper-Inducible GAL Regulation System (CuIGR) in *Saccharomyces cerevisiae*. ACS Synth. Biol..

[69] Knogge W., Schmelzer E., Weissenböck G. (1986). The role of chalcone synthase in the regulation of flavonoid biosynthesis in developing oat primary leaves. Arch. Biochem. Biophys..

[70] Zhou S., Liu P., Chen J., Du G., Li H., Zhou J. (2016). Characterization of mutants of a tyrosine ammonia-lyase from *Rhodotorula glutinis*. Appl. Microbiol. Biotechnol..

[71] Liu D., Sica M.S., Mao J., Chao L.F., Siewers V. (2022). A p-Coumaroyl-CoA Biosensor for Dynamic Regulation of Naringenin Biosynthesis in *Saccharomyces cerevisiae*. ACS Synth. Biol..

[72] Terán W., Felipe A., Segura A., Rojas A., Ramos J.L., Gallegos M.T. (2003). Antibiotic-dependent induction of *Pseudomonas putida* DOT-T1E TtgABC efflux pump is mediated by the drug binding repressor TtgR. Antimicrob. Agents Chemother..

[73] Espinosa-Urgel M., Serrano L., Ramos J.L., Fernández-Escamilla A.M. (2015). Engineering Biological Approaches for Detection of Toxic Compounds: A New Microbial Biosensor Based on the *Pseudomonas putida* TtgR Repressor. Mol. Biotechnol..

[74] Xiong D., Lu S., Wu J., Liang C., Wang W., Wang W., Jin J.M., Tang S.Y. (2017). Improving key enzyme activity in phenylpropanoid pathway with a designed biosensor. Metab. Eng..

[75] Zhou Z., Zhang X., Wu J., Li X., Li W., Sun X., Wang J., Yan Y., Shen X., Yuan Q. (2022). Targeting cofactors regeneration in methylation and hydroxylation for high level production of Ferulic acid. Metab. Eng..

[76] Lee C., Kim I., Lee J., Lee K.L., Min B., Park C. (2010). Transcriptional activation of the aldehyde reductase YqhD by YqhC and its implication in glyoxal metabolism of *Escherichia coli* K-12. J. Bacteriol..

[77] Merchel Piovesan Pereira B., Adil Salim M., Rai N., Tagkopoulos I. (2021). Tolerance to Glutaraldehyde in *Escherichia coli* Mediated by Overexpression of the Aldehyde Reductase YqhD by YqhC. Front. Microbiol..

[78] Turner P.C., Miller E.N., Jarboe L.R., Baggett C.L., Shanmugam K.T., Ingram L.O. (2011). YqhC regulates transcription of the adjacent *Escherichia coli* genes *yqhD* and *dkgA* that are involved in furfural tolerance. J. Ind. Microbiol. Biotechnol..

[79] Verma R., Ellis J.M., Mitchell-Koch K.R. (2021). Dynamic Preference for NADP/H Cofactor Binding/Release in *E. coli* YqhD Oxidoreductase. Molecules.

[80] Kern D., Lapointe J. (1981). The catalytic mechanism of glutamyl-tRNA synthetase of *Escherichia coli*. A steady-state kinetic investigation. Eur. J. Biochem..

[81] Moe J.G., Piszkiewicz D. (1979). Isoleucyl transfer ribonucleic acid synthetase. Steady-state kinetic analysis. Biochemistry.

[82] Yang D., Kim W.J., Yoo S.M., Choi J.H., Ha S.H., Lee M.H., Lee S.Y. (2018). Repurposing type III polyketide synthase as a malonyl-CoA biosensor for metabolic engineering in bacteria. Proc. Natl. Acad. Sci. USA.

[83] Balibar C.J., Walsh C.T. (2006). In vitro biosynthesis of violacein from L-tryptophan by the enzymes VioA-E from *Chromobacterium violaceum*. Biochemistry.

[84] Yang D., Park S.Y., Lee S.Y. (2021). Production of Rainbow Colorants by Metabolically Engineered *Escherichia coli*. Adv. Sci..

[85] Lim H.G., Jang S., Jang S., Seo S.W., Jung G.Y. (2018). Design and optimization of genetically encoded biosensors for high-throughput screening of chemicals. Curr. Opin. Biotechnol..

[86] Lim H.G., Rychel K., Sastry A.V., Bentley G.J., Mueller J., Schindel H.S., Larsen P.E., Laible P.D., Guss A.M., Niu W. (2022). Machine-learning from *Pseudomonas putida* KT2440 transcriptomes reveals its transcriptional regulatory network. Metab. Eng..

[87] Chubukov V., Gerosa L., Kochanowski K., Sauer U. (2014). Coordination of microbial metabolism. Nat. Rev. Microbiol..

[88] Camacho D.M., Collins K.M., Powers R.K., Costello J.C., Collins J.J. (2018). Next-Generation Machine Learning for Biological Networks. Cell.

[89] Hwang H.G., Milito A., Yang J.S., Jang S., Jung G.Y. (2023). Riboswitch-guided chalcone synthase engineering and metabolic flux optimization for enhanced production of flavonoids. Metab. Eng..

[90] Kortmann M., Mack C., Baumgart M., Bott M. (2019). Pyruvate Carboxylase Variants Enabling Improved Lysine Production from Glucose Identified by Biosensor-Based High-Throughput Fluorescence-Activated Cell Sorting Screening. ACS Synth. Biol..

[91] Yao J., He Y., Su N., Bharath S.R., Tao Y., Jin J.M., Chen W., Song H., Tang S.Y. (2020). Developing a highly efficient hydroxytyrosol whole-cell catalyst by de-bottlenecking rate-limiting steps. Nat. Commun..

[92] Ye D.Y., Noh M.H., Moon J.H., Milito A., Kim M., Lee J.W., Yang J.S., Jung G.Y. (2022). Kinetic compartmentalization by unnatural reaction for itaconate production. Nat. Commun..

[93] Seok J.Y., Yang J., Choi S.J., Lim H.G., Choi U.J., Kim K.J., Park S., Yoo T.H., Jung G.Y. (2018). Directed evolution of the 3-hydroxypropionic acid production pathway by engineering aldehyde dehydrogenase using a synthetic selection device. Metab. Eng..

[94] Raghavan S.S., Chee S., Li J., Poschmann J., Nagarajan N., Jia Wei S., Verma C.S., Ghadessy F.J. (2019). Development and application of a transcriptional sensor for detection of heterologous acrylic acid production in *E. coli*. Microb. Cell Factories.

[95] Xu X., Li X., Liu Y., Zhu Y., Li J., Du G., Chen J., Ledesma-Amaro R., Liu L. (2020). Pyruvate-responsive genetic circuits for dynamic control of central metabolism. Nat. Chem. Biol..

[96] Chappell J., Westbrook A., Verosloff M., Lucks J.B. (2017). Computational design of small transcription activating RNAs for versatile and dynamic gene regulation. Nat. Commun..

[97] de Jongh R.P.H., van Dijk A.D.J., Julsing M.K., Schaap P.J., de Ridder D. (2020). Designing Eukaryotic Gene Expression Regulation Using Machine Learning. Trends Biotechnol..

